# Phytoestrogenic Effects of Blackcurrant Anthocyanins Increased Endothelial Nitric Oxide Synthase (eNOS) Expression in Human Endothelial Cells and Ovariectomized Rats

**DOI:** 10.3390/molecules24071259

**Published:** 2019-03-31

**Authors:** Kayo Horie, Naoki Nanashima, Hayato Maeda

**Affiliations:** 1 Department of Bioscience and Laboratory Medicine, Hirosaki University Graduate School of Health Sciences, 66-1 Hon-cho, Hirosaki, Aomori 036-8564, Japan; nnaoki@hirosaki-u.ac.jp; 2 Faculty of Agriculture and Life Science, Hirosaki University, 3 Bunkyo-cho, Hirosaki, Aomori 036-8561, Japan; hayatosp@hirosaki-u.ac.jp

**Keywords:** anthocyanin, blackcurrant extract, endothelial nitric oxide synthase (eNOS), phytoestrogen, vascular endothelial cells

## Abstract

Phytoestrogens are plant-derived chemicals that are found in many foods and have estrogenic activity. We previously showed that blackcurrant extract (BCE) and anthocyanins have phytoestrogenic activity mediated via estrogen receptors (ERs), and anthocyanins may improve vascular function. BCE contains high levels of anthocyanins, but their health-promoting effects are unclear. This study examined the effects of BCE on the regulation of endothelial nitric oxide synthase (eNOS) expression and nitric oxide (NO) synthesis in human endothelial cells as key regulators in cardiovascular disease. The results showed that eNOS mRNA levels were significantly upregulated in BCE- or anthocyanin-treated human vascular endothelial cells but decreased in cells treated with fulvestrant, an ER antagonist. These results corresponded with NO levels, suggesting that BCE and anthocyanin may regulate NO synthesis via eNOS expression. Thus, the phytoestrogenic effects exerted by BCE via ERs influenced eNOS mRNA expression and NO synthesis. In vivo, we investigated whether anthocyanin-rich BCE upregulated eNOS protein expression in ovariectomized (OVX) rats, a widely used animal model of menopause. Our results showed that anthocyanin-rich BCE significantly upregulated eNOS mRNA levels and NO synthesis through phytoestrogenic activity and therefore promoted blood vessel health in OVX rats as a postmenopausal model.

## 1. Introduction

The risk of cardiovascular disease (CVD) generally increases with age but is lower in premenopausal women than in men of similar age; the risk increases markedly in women after menopause [1,2]. This phenomenon is presumably in part due to estrogen deficiency in postmenopausal women, and the increased risk of CVD is associated with vascular endothelial dysfunction [2,3,4].

Hormone-replacement therapy (HRT) containing estrogen is the most effective treatment for menopausal symptoms in healthy women. However, when administering estrogen treatments, the risk of venous thrombosis and breast cancer must also be considered [5,6] As longevity continues to improve, the number of women with menopausal symptoms will likely rise. Therefore, safe and effective non-hormonal treatments for menopausal symptoms are needed.

Phytoestrogens are plant-derived chemicals that have estrogenic activity mediated by estrogen receptors (ERs), which initiate estrogen-dependent transcription [7]. Phytoestrogens are reported to have an effect at least 1000–10,000 times less potent than that of estradiol [5]. Thus, phytoestrogens can potentially be used as an alternative to HRT.

Anthocyanins, such as cyanidin and delphinidin, are plant pigments belonging to the flavonoid family and may exhibit phytoestrogenic activity [8]. Blackcurrants are reported to contain four anthocyanins: cyanidin-3-glucoside (C3G), cyanidin-3-rutinoside (C3R), delphinidin-3-glucoside (D3G), and delphinidin-3-rutinoside (D3R) [9]; D3R and C3R are anthocyanins specific to blackcurrant [10]. These anthocyanins are known to have many health benefits, such as suppression of inflammation [11,12,13], improvement of glucose metabolism [14,15,16] and promotion of cardiovascular health [17,18,19]. Furthermore, we previously reported that blackcurrant anthocyanins act as phytoestrogens both in vitro and in vivo [9,13,20,21].

Nitric oxide (NO) synthesis is known to play a key role in regulating vascular function and health and represents one of the most well-established vascular effects of estrogen [22,23]. NO is a pivotal vasoprotective molecule and is released from endothelial cells via endothelial nitric oxide synthase (eNOS) [24]; estradiol mediates eNOS activation via interaction with ERs [25,26].

A major goal of our study was to clarify the beneficial effects of an anthocyanin-rich blackcurrant extract (BCE) and four blackcurrant anthocyanins on vascular health. For this purpose, we exposed human vascular endothelial cells including human umbilical vein endothelial cells (HUVECs) and EA.hy926 cells to BCE and anthocyanins and subsequently assessed changes in eNOS gene expression using microarrays and quantitative polymerase chain reaction (qPCR), followed by quantitation of NO release using a Nitric Oxide Assay Kit. Furthermore, we evaluated the expression of eNOS protein in blood vessels of ovariectomized (OVX) rats, which are estrogen-deficient. Collectively, our results suggest that the anthocyanin-rich BCE may promote blood vessel health.

## 2. Results and Discussion

### 2.1. Microarray Gene Expression Profiling of HUVECs Exposed to BCE

We initially compared gene expression in HUVECs before and after exposure to 0.5, 1.0, and 2.0 μg/mL BCE using microarrays. BCE at 2.0 μg/mL was found to induce the highest level of gene expression. Thus, we performed Ingenuity Pathway Analysis (IPA) of array data corresponding to 2.0 μg/mL BCE exposure to investigate the functional relationships between sets of genes showing altered expression levels. BCE exposure upregulated the expression of some beneficial genes related to the vasculature, and several predicted upstream regulators were detected, such as *ESR1*, raloxifene, tretinoin, and NO (Table 1).

The z-score of ERα was 2.7, and expression changes were assessed for the gene sets regulated by ERα (Table S1). In our previous studies, BCE was shown to exert phytoestrogenic activity mediated via ERα in human breast cancer cells (MCF7) [9] and human fibroblasts (TIG113 cells) [21]. In addition, several studies reported that ERα is more highly expressed in endothelial cells than ERβ and plays a more important role in mediating the effects of estrogen in the vascular endothelium [27,28]. Furthermore, a preliminary experiment performed in our laboratory demonstrated that, in HUVECs, ERα expression was about 10 times higher than that of Erβ (data not shown). These results suggest that the phytoestrogenic effects of BCE on HUVECs require ERα, which is consistent with a previous report [9,21].

Raloxifene showed a z-score of 2.3, and genes downstream of raloxifene were upregulated by BCE. Raloxifene is a tissue-selective ER modulator (SERM) clinically effective for the prevention of postmenopausal osteoporosis. Raloxifene is known to behave similarly to estrogen and has been shown to induce NO production by ER-dependent enhancement of eNOS enzymatic activity in human endothelial cells [29,30,31]. Therefore, these results suggest that BCE acted similarly to raloxifene in HUVECs.

The z-score of NO was 2.1, and the expression changes associated with the gene sets regulated by estradiol and NO were assessed (Table S2). These results indicate that BCE significantly altered the expression levels of genes involved in the pathway related to the synthesis of NO.

### 2.2. eNOS mRNA Expression in Human Endothelial Cells Determined by Reverse Transcription (RT)-qPCR Analysis

**Figure 1:**
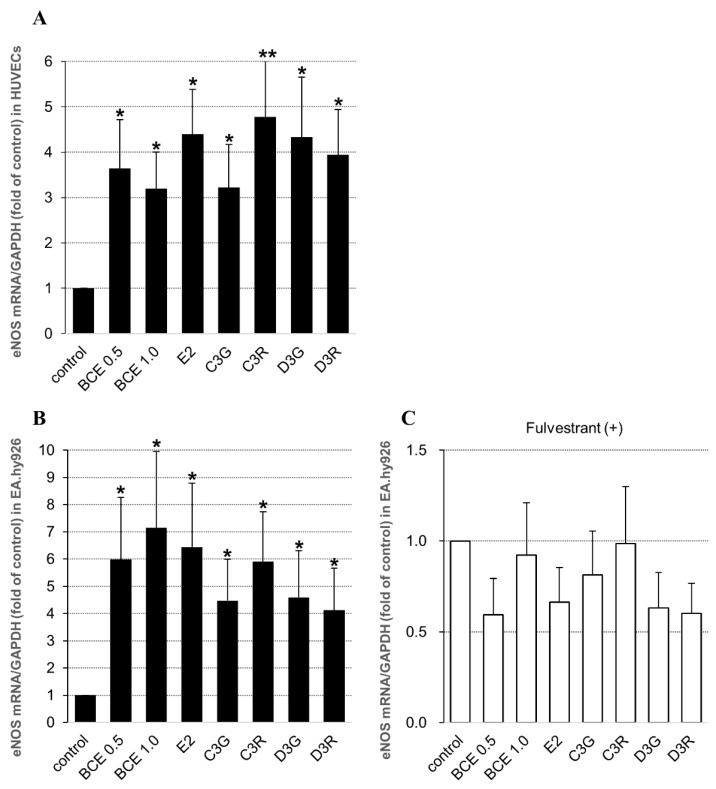
Effects of blackcurrant extract (BCE), anthocyanins, and 17β-estradiol (E2) on endothelial nitric oxide synthase (eNOS) mRNA expression levels in: human vascular endothelial cells (HUVECs) (**A**); and EA.hy926 cells (**B**,**C**). Cells were treated with 0.5 or 1.0 μg/mL BCE, 10 μM anthocyanin, or 10 nM E2 for 24 h and in the presence of 100 nM fulvestrant for 24 h in the case of EA.hy926 cells (**C**). Data are shown as the mean ± standard error of the mean (SEM) of at least three independent experiments. * *p* < 0.05, ** *p* < 0.01 vs. untreated control cells. C3G, cyanidin-3-glucoside; C3R, cyanidin-3-rutinoside; D3G, delphinidin-3-glucoside; D3R, delphinidin-3-rutinoside.

To determine the effects of anthocyanin or BCE on the expression of eNOS, RT-qPCR analysis was performed. eNOS mRNA levels were significantly upregulated in BCE- or anthocyanin-treated HUVECs (Figure 1A), and, to confirm this effect in other endothelial cells, we performed the same experiment using EA.hy926 cells. The results were consistent with those of HUVECs (Figure 1B). eNOS mRNA expression increased with BCE treatment at both 0.5 and 1.0 μg/mL dose-independently. The expression levels were not significantly different between BCE concentrations, and it appeared that the optimal concentration of BCE depended on cell type. In previous studies, the effective concentration of BCE was found to be approximately 1 μg/mL [9,13,20,21]. In addition, cell exposure to all anthocyanins included in BCE significantly increased eNOS mRNA levels, but there were no significant differences among the four anthocyanins.

On the other hand, in fulvestrant-treated cells, eNOS mRNA expression decreased but was not significantly different compared with control levels (Figure 1C). Fulvestrant is a synthetic ER antagonist; therefore, we speculated that BCE exerts phytoestrogenic activity mediated via ERs, which impacts eNOS expression.

### 2.3. NO Synthesis in Human Endothelial Cells

**Figure 2:**
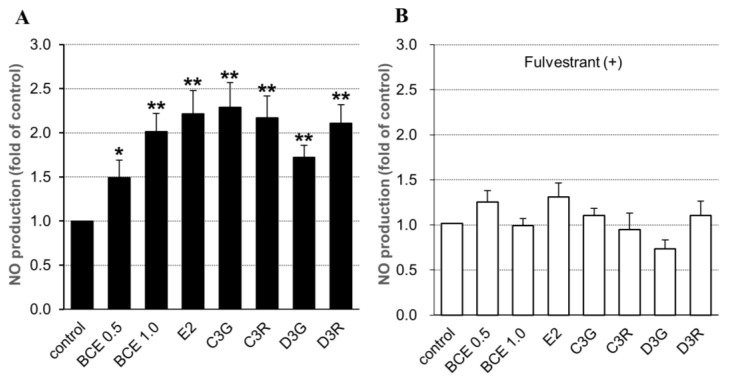
Effects of BCE, anthocyanins, and E2 on absolute nitrite and/or nitrate levels in supernatants of EA.hy926 cells. Cells were treated with 0.5 or 1.0 μg/mL BCE, 10 μM anthocyanin, or 10 nM E2 in the absence (**A**) or presence (**B**) of 100 nM fulvestrant for 24 h. Culture supernatants were collected after five days of incubation. Data are shown as the mean ± SEM of at least three independent experiments. * *p* < 0.05, ** *p* < 0.01 vs. untreated control cells.

We next investigated whether BCE or anthocyanins stimulated NO synthesis in EA.hy926 cells following five days of incubation in the presence or absence of fulvestrant for 24 h. NO levels were significantly higher in BCE- or anthocyanin-treated cells than in control cells; there were no significant differences between BCE concentrations and among the four kinds of anthocyanins (Figure 2A). On the other hand, in fulvestrant-treated cells, NO synthesis levels were decreased but not significantly compared with control levels (Figure 2B). These results corresponded with eNOS mRNA expression, which suggested that BCE and anthocyanin may regulate NO synthesis via eNOS expression.

Red wine polyphenols increased eNOS expression and subsequent endothelial NO release in HUVECs [32]. In particular, resveratrol, a type of red wine polyphenol, induced eNOS activation and NO production in HUVECs [33]. Genistein, a soy phytoestrogen, enhanced the expression of eNOS mRNA and protein and subsequently elevated NO synthesis in human endothelial cells [34]. Furthermore, artichoke flavonoids upregulated the gene expression of eNOS in human endothelial cells [35]. However, the activation of eNOS expression by BCE has been rarely reported [17,36], but our results are consistent with these previous reports.

### 2.4. NO Synthesis-Related Gene Expression in HUVECs Exposed to BCE

The whole-transcript microarray analysis of HUVECs exposed to BCE (2.0 µg/mL) showed that expression of NO-related genes *VEGFA*, *HSP90AB1*, *PIK3CA*, and *AKT1* was upregulated (Table 2), indicating that BCE altered the pathways related to NO synthesis.

The expression of the *VEGFA* gene was upregulated by 1.7-fold based on microarray results, although the expression of other *VEGF* genes was not substantially altered. *VEGFC* expression was upregulated by 1.2-fold, and *VEGFB* expression was not different from control levels (data not shown). Previous studies showed that VEGF is associated with eNOS expression [37,38]; in addition, it has been shown that stimulation of HUVECs with VEGFA results in a rapid release of NO and upregulation of eNOS expression [39]. Furthermore, VEGF has been shown to activate phosphatidylinositol 3-kinase (PI3K), leading to phosphorylation of Akt, which phosphorylates eNOS, thereby increasing eNOS enzymatic activity [40].

BCE may regulate VEGF expression, thereby promoting NO release, through mechanisms involving tyrosine and PI3K kinases. In this study, the levels of *PIK3CA* (1.4-fold difference) and *AKT1* (1.1-fold difference) gene expression based on microarray were not significantly upregulated (Table 2). In addition, we investigated PI3K and phospho-Akt protein expression by immunohistochemical staining of blood vessel endothelial cells from BCE-treated OVX rats. However, neither protein was clearly detected (data not shown). It is possible that VEGF promotes NO release through mechanisms involving tyrosine and PI3K kinases in the early phase of stimulation [37].

Based on the microarray, heat shock protein 90 (*HSP90*) expression was upregulated by 1.4-fold. HSP90 is a molecular chaperone that enhances the activation of eNOS [41], and delayed NO production mediated by VEGF is dependent on eNOS phosphorylation induced by intracellular mediators such as Hsp90 and Akt [42]. Thus, our study suggested that BCE may trigger NO synthesis in endothelial cells not only through phytoestrogenic activity but also via other pathways.

### 2.5. eNOS Protein Expression in BCE-Treated OVX Rats Determined by Immunohistochemical Staining

**Figure 3:**
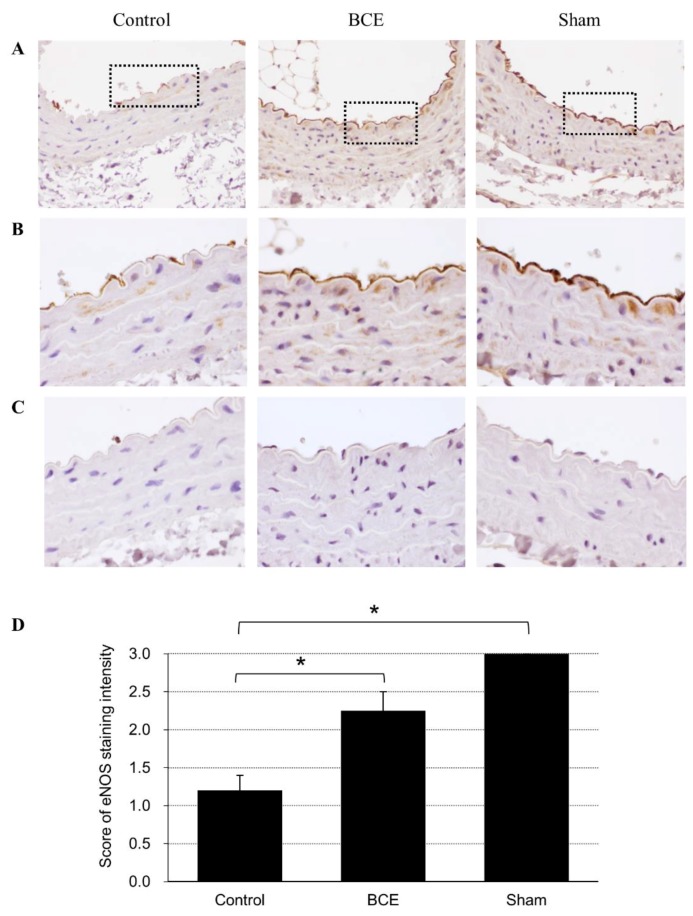
Immunohistochemical staining of eNOS in control (ovariectomized (OVX) + 0% BCE), BCE (OVX + 3% BCE), and sham surgery rats. Images of eNOS protein expression at lower magnification (×200) (**A**). Images of the boxed areas shown in (**A**) at ×400 magnification (**B**). Tissues not treated with primary antibody were used as negative controls (**C**). Scoring of the staining intensity of eNOS (**D**). Data are shown as the mean score ± standard error. * *p* < 0.05.

To verify the in vitro effects of BCE on eNOS protein expression in vivo, we fed OVX rats a diet containing 3% BCE. These rats do not produce estrogen and therefore are considered a model of menopause. We assessed whether dietary BCE increased the expression of eNOS protein by immunohistochemical staining of blood vessel endothelial cells from the model rats (Figure 3). eNOS protein expression was significantly higher in OVX rats treated with 3% BCE and in sham surgery rats than in untreated OVX rats (control). Thus, our results showed that dietary intake of BCE increased eNOS protein levels in the blood vessels of OVX rats, which is in agreement with the in vitro results.

Our results showed that dietary intake of BCE increased eNOS protein levels in the blood vessels of OVX rats. Interestingly, BCE-treated endothelial cells showed upregulation of some beneficial genes in the vasculature, and several predicted upstream regulators were detected (Table 1). Of these, raloxifene is a SERM, and BCE may have estrogenic activity similar to that of raloxifene. Furthermore, tretinoin is known as an all-trans-retinoic acid (ATRA), a naturally occurring derivative of vitamin A (retinol). Retinoids such as tretinoin are important regulators of cell division and proliferation. Its cosmetic effects on the skin are well known, and several studies have demonstrated the effects of tretinoin on blood vessels [43,44,45]. The current results suggest that BCE may exert an effect similar to that of tretinoin on HUVECs. Thus, the synergistic effects of the phytoestrogenic activity and other components of BCE may be beneficial for vessel health in postmenopausal women.

## 3. Materials and Methods

### 3.1. Materials and Cell Culture

The BCE powder CaNZac-35 was purchased from Koyo Mercantile Co. (Tokyo, Japan). Our previous study showed that BCE contains high concentrations of polyphenols (37.6 g/100 g BCE) and anthocyanins C3G (5.6%), C3R (32.0%), D3G (16.8%), and D3R (45.3%) [9]. C3G, C3R, D3G, and D3R were purchased from Nagara Science (Gifu, Japan). 17β-Estradiol (E2) and fulvestrant (ICI 182,780) were purchased from Sigma-Aldrich (St. Louis, MO, USA).

We used two kinds of human vascular endothelial cells, primary HUVECs and immortalized endothelial cells (EA.hy926). HUVECs and EA.hy926 cells were obtained from PromoCell (Heidelberg, Germany) and American Type Culture Collection (Manassas, VA, USA), respectively. We used cell culture medium without phenol red and supplemented with charcoal-stripped fetal bovine serum (FBS) to elucidate the effects of estrogen and phytoestrogen on the cells. Specifically, HUVECs were cultured in Endothelial Cell Basal Medium 2 phenol red-free (TaKaRa, Tokyo, Japan) supplemented with SupplementMix (TaKaRa), and the EA.hy926 cells were cultured in Dulbecco’s modified Eagle’s medium (D-MEM)/Ham’s F-12 with l-glutamine and sodium pyruvate (Wako Pure Chemical Industries Ltd., Osaka, Japan) supplemented with 10% charcoal-treated FBS (Thermo Fisher Scientific, Tokyo, Japan) and 1% penicillin–streptomycin (Thermo Fisher Scientific). All culture experiments were conducted at 37 °C in a humidified incubator containing 5% CO_2_.

### 3.2. Microarray Gene Expression Profiling

Sample preparation for microarray was performed according to our previous study [21]. HUVECs were seeded in 21-mm^2^ culture dishes until they reached confluence. The medium was changed, and then the cells were treated with BCE (2.0 μg/mL) or left untreated. After a 24-h incubation period, the cells were washed twice with phosphate-buffered saline (PBS), and total RNA was extracted using the RNeasy mini kit (Qiagen, Hilden, Germany). RNA labeling and hybridization were performed using the Agilent One-Color Microarray-Based Gene Expression Analysis protocol (Santa Clara, CA, USA). Briefly, 100 ng of total RNA from each sample was linearly amplified and labeled with Cy3-dCTP. The resultant labeled cRNAs were purified using the RNeasy mini kit (Qiagen). Labeled and fragmented cRNA was hybridized to a SurePrint G3 Human Gene Expression microarray (8 × 60 K version 3; Agilent Technologies). Labeling, hybridization, image scanning, and data analysis were performed at Macrogen Japan Corp. (Tokyo, Japan). The microarray dataset is available at http://www.ncbi.nlm.nih.gov/geo, under the accession code GSE121730.

### 3.3. IPA

Genes up- or downregulated by ≥1.5-fold in HUVECs following exposure to 2.0 μg/mL BCE were analyzed using IPA software (version 36601845). The z-score algorithm was utilized to reduce the possibility of false-positive results, where z ≥ 2.0 indicated that transcript expression was significantly increased, and z ≤ −2.0 indicated that expression was significantly decreased.

### 3.4. RT-qPCR

eNOS mRNA expression was evaluated with RT-qPCR analysis as previously described [21]. Briefly, HUVECs and EA.hy926 cells were seeded in a 12-well culture plate and cultured until confluent. The medium was changed, and then cells were treated with or without anthocyanins (10 μM), BCE (0.5 or 1.0 μg/mL), or E2 (10 nM), in the presence or absence of 100 nM fulvestrant, for 24 h. The cells were incubated for an additional 24 h and then washed twice with PBS. Total RNA was extracted using the RNeasy mini kit (Qiagen). cDNA was reverse-transcribed from total RNA (200 ng) using the PrimeScript ^®^ RT Master Mix (TaKaRa). Levels of specific mRNAs were quantified by qPCR using SYBR^®^ Premix ExTaq™ II (TaKaRa). PCR amplification consisted of 30 s at 95 °C, 5 s at 95 °C, and 30 s at 60 °C for 40 cycles. Transcript levels were normalized to those of glyceraldehyde 3-phosphate dehydrogenase (*GAPDH*). The primers were as follows (5′→3′): eNOS, forward, GACATTGAGAGCAAAGGGCTGC; reverse, CGGCTTGTCACCTCCTGG [34] and GAPDH, forward, TGAGAACGGGAAGTCTGTCA; reverse, TCTCCATGGTGGTGAAGACG.

PCR specificity was verified by melting curve analysis. All samples were analyzed in triplicate, and relative gene expression was calculated by the 2^−ΔΔCt^ method.

### 3.5. NO Measurement

EA.hy926 cells were seeded in a 12-well culture plate and cultured until confluent. The medium was changed, and then the cells were incubated with or without anthocyanins (10 μM), BCE (0.5 or 1.0 μg/mL), or E2 (10 nM), in the presence or absence of 100 nM fulvestrant, for 24 h. After the cells were incubated for another five days, the culture media were collected and concentrated with a SpeedVac concentrator (SPD1010, Thermo Fisher Scientific, Tokyo, Japan). NO release was quantitated using the QuantiChrom ^TM^ Nitric Oxide Assay Kit (Funakoshi, Tokyo, Japan) according to the manufacturer’s manual. Briefly, culture media were deproteinated with ZnSO_4_ and NaOH, followed by the addition of Working Reagent from the kit and incubation for 10 min at 60 °C. Samples were transferred to a 96-well plate, and then the optical density (OD) was measured with a microplate reader (Benchmark, Bio-Rad, CA, USA) at a wavelength of 570 nm. Data were converted into concentrations based on standard curves constructed with a standard sample.

### 3.6. Animals and Treatments

Female Sprague-Dawley rats (12 weeks old) were purchased (CLEA Japan, Inc., Tokyo, Japan) and group-housed in plastic cages in air-conditioned rooms with a 12-h light/dark cycle at the Institute for Animal Experiments of Hirosaki University Graduate School of Medicine. All rats had free access to water and food. The Animal Research Committee of Hirosaki University approved this study (permission number: G16004), which was conducted in accordance with the rules for animal experimentation of Hirosaki University. Our previous studies showed that 3% BCE exerts phytoestrogenic effects in rats [9]. In this study, all of the rats received the AIN-93M diet (Oriental Yeast Co., Ltd., Tokyo, Japan), with or without 3% BCE, and were divided into three groups and treated as described previously [21]: (1) OVX rats treated with 3% BCE for 3 months (n = 3); (2) OVX rats receiving no BCE treatment (control) (n = 4); and (3) sham surgery rats receiving no BCE treatment (n = 3). At the end of the experiment, the animals were euthanized, and the celiac artery was removed.

### 3.7. Immunohistochemical Staining of eNOS Protein

Celiac artery tissue was fixed in 10% formalin neutral buffer solution and paraffin-embedded into tissue blocks. Serial 3-μm-thick sections were cut and placed on glass slides for immunohistochemical staining using an EnVision^TM^ detection system (DakoCytomation A/S, Glostrup, Denmark) according to the manufacturer’s recommendations. Endogenous peroxidases in the specimens were blocked with Peroxidase-Blocking Solution (DakoCytomation A/S) for 5 min at room temperature, and after incubation with Protein Block Serum-Free reagent (DakoCytomation A/S), an anti-eNOS antibody (prediluted; Abcam, Tokyo, Japan) was applied as the primary antibody. Following incubation for 90 min at room temperature and washing, the tissues were incubated with EnVision^TM^/HRP Rabbit/Mouse secondary antibodies (DakoCytomation A/S) for 30 min at room temperature and then with the chromogen 3,3′-diaminobenzidine. Nuclei were counterstained using Mayer’s hematoxylin (Wako Pure Chemical Industries Ltd.). Tissue sections that were not treated with primary antibody were used as negative controls. The staining intensities of the tissue samples were determined semi-quantitatively (none, 0; weak, 1; moderate, 2; intense, 3).

### 3.8. Statistical Analysis

Results are expressed as the mean ± standard error of the mean (SEM) of at least three independent experiments. Statistical analyses were performed using BellCurve for Excel ver. 2.13 software (Social Survey Research Information, Tokyo, Japan) and Kruskal–Wallis analysis with the Steel post hoc test; *p* < 0.05 was considered to indicate statistical significance.

## 4. Conclusions

The present study demonstrated that the phytoestrogenic activity of blackcurrant anthocyanins and BCE strongly increased eNOS mRNA expression and NO production in human endothelial cells. Furthermore, dietary BCE increased eNOS protein expression in an OVX rat model, confirming the biological relevance of the in vitro findings. These results suggest that anthocyanin-rich BCE may have beneficial health effects on blood vessels in postmenopausal women. In this study, we did not administer BCE to humans, given that the eNOS protein is critical for maintaining vascular integrity; however, we intend to perform clinical studies in the future.

## Figures and Tables

**Table 1:** IPA of blackcurrant extract (BCE)-treated human umbilical vein endothelial cells (HUVECs).

Predicted Upstream Regulator	z-Score
ESR1 (ERα)	2.7
Raloxifene	2.3
Tretinoin	2.2
Nitric oxide (NO)	2.1

Upstream analysis of HUVECs treated with 2.0 μg/mL BCE. In the z-score algorithm, z ≥ 2.0 indicated a significant increase in transcript expression.

**Table 2:** Expression of NO synthesis-related genes in BCE-treated HUVECs.

Gene Symbol	Gene Name	* Fold Change	Accession No.
*VEGFA*	Vascular endothelial growth factor A	1.7	NM_001025366
*HSP90AB1*	Heat shock protein 90kDa alpha (cytosolic), class B member 1	1.4	NM_007355
*PIK3CA*	Phosphatidylinositol-4,5-bisphosphate 3-kinase, catalytic subunit alpha	1.4	NM_006218
*AKT1*	v-Akt murine thymoma viral oncogene homolog 1	1.1	NM_005163

* Alterations of mRNA expression in HUVECs treated with BCE (2.0 µg/mL for 24 h) determined by microarray analysis.

## Supplementary Materials

The following are available online at https://www.mdpi.com/1420-3049/24/7/1259/s1, Table S1: Effects of BCE on ESR1 (ERα)-regulated gene expression in HUVECs, Table S2: Effects of BCE on nitric oxide-regulated gene expression in HUVECs.
