# Supplementary figures and images for: Phytoestrogenic Effects of Blackcurrant Anthocyanins Increased Endothelial Nitric Oxide Synthase (eNOS) Expression in Human Endothelial Cells and Ovariectomized Rats
Source: Molecules. 2019 Mar 31;24(7):1259. doi: 10.3390/molecules24071259 (PMC6480453; doi:10.3390/molecules24071259)

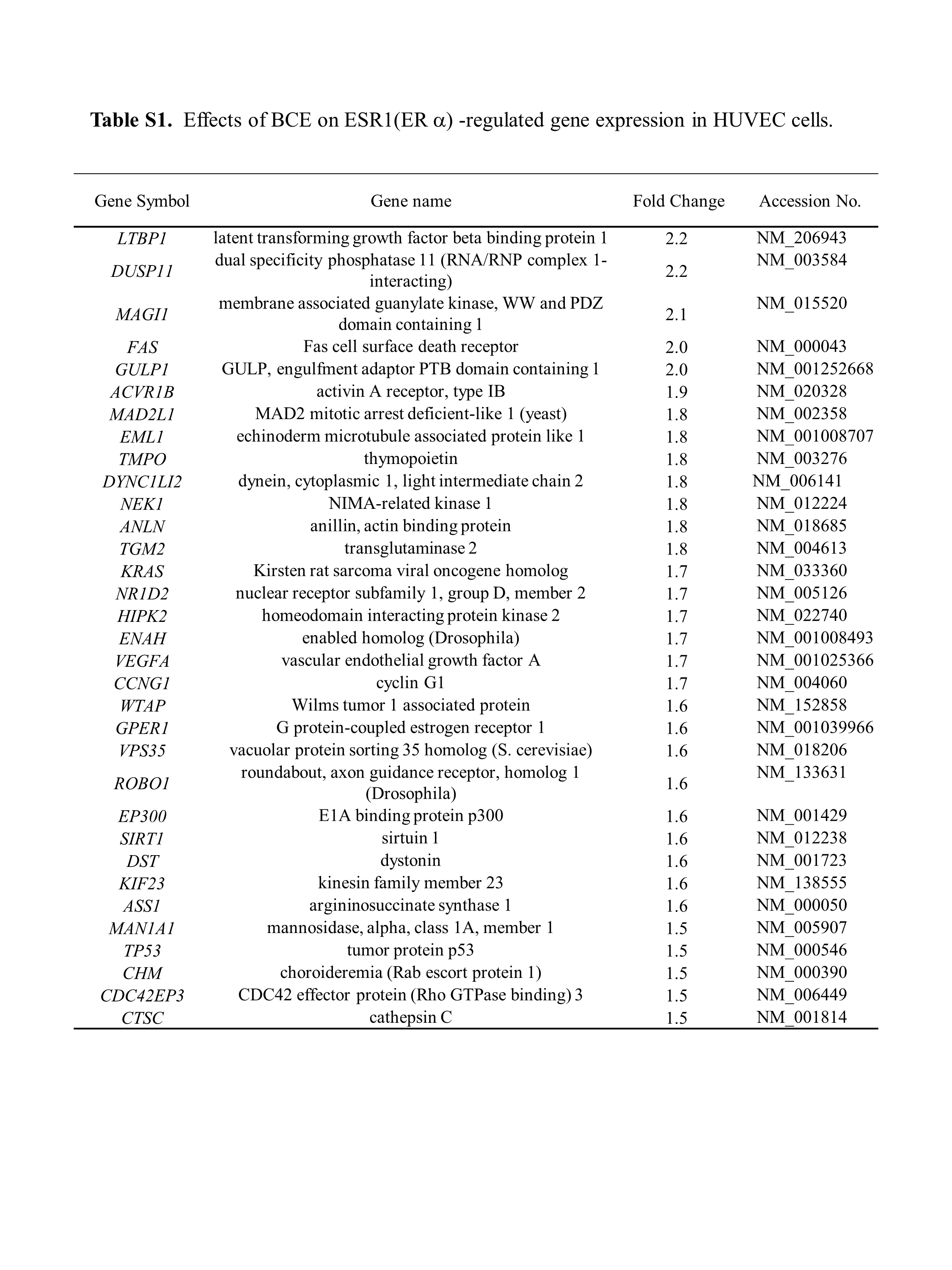

Supplement: Supplementary file 1 [file molecules-24-01259-s001.zip › Table S1_300dpi.tif]

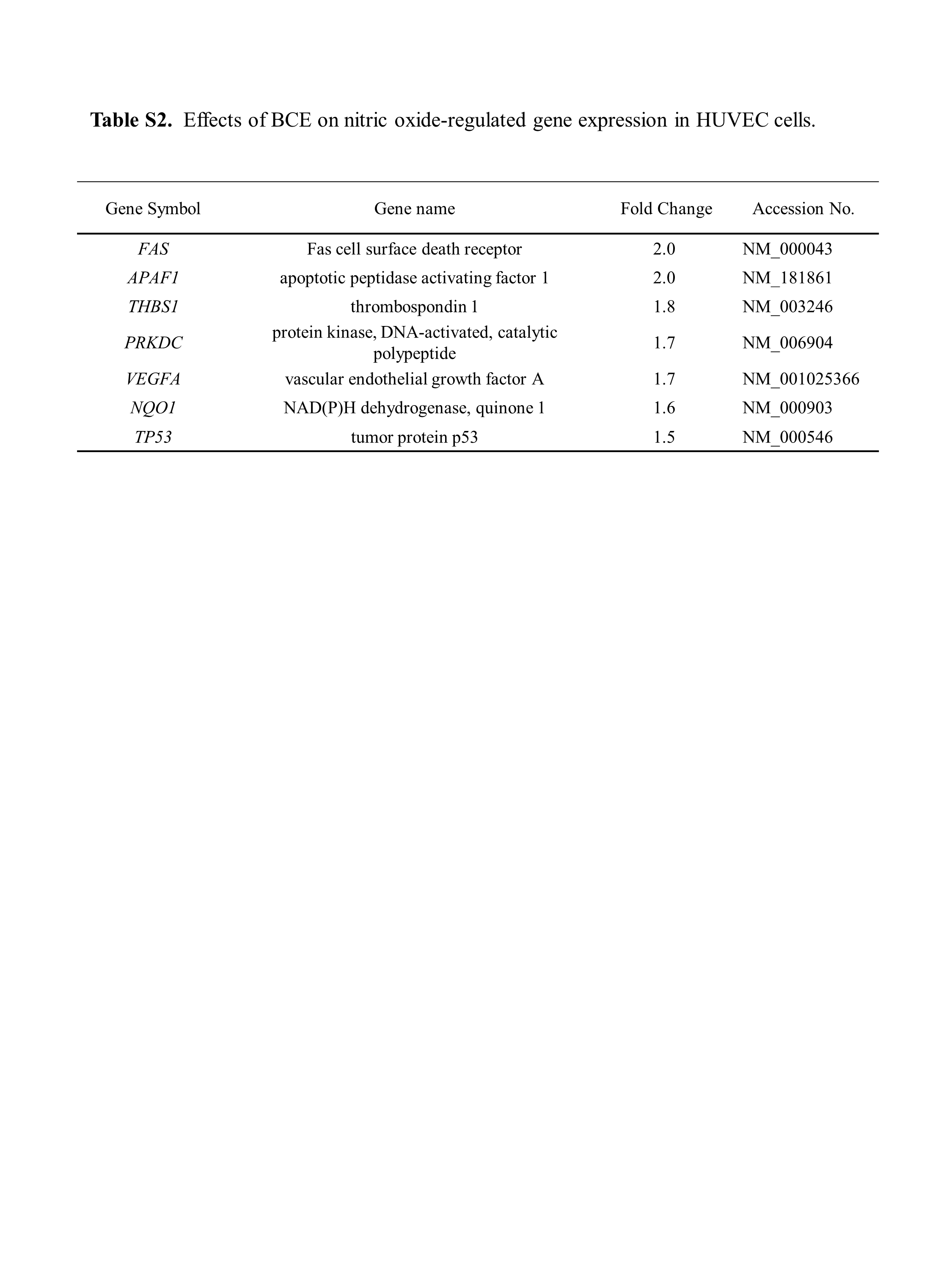

Supplement: Supplementary file 1 [file molecules-24-01259-s001.zip › Table S2_300dpi.tif]
